# Valorization of Polymethylmethacrylate Scrap Reinforced with Nano Carbon Black with Optimized Ratio in Extrusion-Based Additive Manufacturing

**DOI:** 10.3390/polym17101383

**Published:** 2025-05-17

**Authors:** Nikolaos Michailidis, Nectarios Vidakis, Constantine David, Dimitrios Sagris, Vassilis M. Papadakis, Apostolos Argyros, Nikolaos Mountakis, Maria Spyridaki, Markos Petousis

**Affiliations:** 1 Physical Metallurgy Laboratory, Mechanical Engineering Department, School of Engineering, Aristotle University of Thessaloniki, 54124 Thessaloniki, Greece; nmichail@auth.gr (N.M.); aargyros@auth.gr (A.A.); 2 Centre for Research & Development of Advanced Materials (CERDAM), Centre for Interdisciplinary Research and Innovation, Balkan Centre, Building B’, 10th km Thessaloniki-Thermi Road, 57001 Thessaloniki, Greece; 3 Department of Mechanical Engineering, Hellenic Mediterranean University, 71410 Heraklion, Greece; vidakis@hmu.gr (N.V.); mountakis@hmu.gr (N.M.); mspyridaki@hmu.gr (M.S.); 4 Department of Mechanical Engineering, International Hellenic University, Serres Campus, 62124 Serres, Greece; david@ihu.gr (C.D.); dsagris@ihu.gr (D.S.); 5 Department of Industrial Design and Production Engineering, University of West Attica, 12243 Athens, Greece; v.papadakis@uniwa.gr; 6 Institute of Electronic Structure and Laser of the Foundation for Research and Technology-Hellas (IESL-FORTH)–Hellas, N. Plastira 100m, 70013 Heraklion, Greece

**Keywords:** polymethylmethacrylate (PMMA), carbon black (CB), three-dimensional (3D) printing, environmental sustainability, material extrusion, recycling, mechanical characterization, additive manufacturing

## Abstract

To promote environmental sustainability, this research investigated the potential of utilizing recycled polymethylmethacrylate (PMMA) as raw material in material extrusion (MEX) additive manufacturing (AM). To enhance its mechanical response, carbon black (CB) was employed as the filler in nanocomposite formation. Filament extrusion of the mixture at different concentrations produced printable feedstocks for MEX AM. Rheological analysis (viscosity and material flow rate) showed that the CB introduction to the matrix was beneficial for consistent layer deposition, while differential scanning calorimetry and thermogravimetric analyses verified the thermal stability of the nanocomposites during processing. Mechanical properties were optimized, with increases in modulus (27.8% and 25.8%, respectively, in tensile and bending loadings) and tensile strength at optimal CB loadings. Dynamic mechanical analysis revealed the viscoelastic response of the nanocomposites. Raman and energy dispersive spectroscopy provided element-related insights. Surface morphology and parts structure were observed employing scanning electron microscopy and micro-computed tomography, respectively, revealing a positive impact on the AM parts due to the CB presence in the nanocomposites. The 4 wt.% in CB content nanocomposite was the optimum one. This research pioneers the development of new sustainable nanocomposite filaments and highlights the potential of next-generation MEX-based AM.

## 1. Introduction

3D printing can be used in applications derived from numerous sectors, such as the medical [1,2,3,4], automotive [5], aviation [6], aerospace [7,8,9], agriculture [10], electronics [11], and even food [12,13] industries [14]. There is a great variety of materials that can be employed in 3D printing, depending on the type of application, namely metals [15], polymers [16], ceramics [17], various composites [18], and smart [19] or special materials [20].

In particular, polymers such as Acrylonitrile Butadiene Styrene (ABS) [21], Polylactic Acid (PLA) [22,23], polypropylene (PP) [24], polycarbonate (PC) [25], polyethylene terephthalate glycol (PETG) [26], polymethylmethacrylate (PMMA) [27,28], and High-Density Polyethylene (HDPE) [29] are selected very often either in neat [29,30,31] or composite [32,33] form, covering a wide range of applications. Inevitably, their extensive utilization can result in waste management issues and environmental consequences, leading to undesirable effects. Recycling is a possible way of treating waste and has been increasingly employed in the available research work [34,35,36]. Consequently, sustainability and circular economy [37,38,39] are promoted by the use of recycled polymers in additive manufacturing (AM) processes [40].

PMMA is an acrylate composed of free radicals as well as chain polymerization of methyl methacrylate [41]. PMMA lightweight material is biocompatible and possesses chemical as well as physical stability [42,43], durability, and ease of mechanical behavior adjustability [44]. It is usually employed for biomedical applications such as dental prostheses [45,46,47,48], palatal fillers, maxillofacial prostheses, eye lenses, and drug delivery [47,49]. Due to its characteristics, it is also popular in solar, optical, sensor, nanotechnology, battery electrolyte, molecular separation, pneumatic actuation, and polymer conductivity technologies [50]. In 3D printing, its eco-friendliness has been investigated in terms of the required energy to build parts in relation to its mechanical properties [51], which have also been investigated [52]. Furthermore, it has been utilized as a matrix material for biomedical composites [53], as this is a field it is often used in. In this publication, it was shown that it can be used as a matrix polymer in composites, without compromising its thermal or other properties, while with the specific filler, multifunctional behavior was enabled, with improved mechanical response and biocidal capabilities. Moreover, it has been reported that it can be used in hybrid AM technologies [54], further expanding its applicability. To the best of the authors’ knowledge, although there are investigations on the recycling of various polymers [55,56,57], there are limited publications on the recycling of PMMA [58,59]. The paper from our research group [59] investigates the recycling of PMMA in MEX AM in unfilled form. This paper reports that PMMA can be thermomechanically reprocessed up to six times, with its mechanical strength in the tensile experiment increasing by 9.2% in the second thermomechanical course. Therefore, it has been reported that recycled PMMA is more than adequate for use as raw material in MEX AM. Nonetheless, there is a need for further extensive investigation, as there could be plenty of benefits, considering both sustainability and potential recycled PMMA applications, for example, by enhancing its properties and specifications through the addition of suitable fillers and the formation of respective composites.

Owing to the constantly increasing use of PMMA, the market size is also rising and is expected to grow even more, considering the numerous available reports [60,61,62,63]. Grand View Research reported that USD 5654.2 million in 2023 could have a 5.3% compound annual growth rate (CAGR) and reach USD 8100 million by 2030 [64]. According to previous research, in the 2024–2034 forecast period, the PMMA market size was measured to be USD 9280 million, up from USD 5430 million (5.5% CAGR) [65]. Then, between 2022 and 2030, a 5.5% CAGR has been calculated, predicting that the USD 4.5 million PMMA market volume in 2021 could increase up to USD 7280 million in 2030 [66].

Based on the above numbers, it can be easily assumed that large amounts of PMMA material will result in waste. Exploiting this waste would be beneficial for the environment and contribute to the sustainability of materials. AM is a technology in which the potential of reusing the PMMA material can be explored, as it is a commonly employed technology for recycling polymer exploitation. The findings presented in the bibliography show strong potential for the reuse of various polymers after they reach the end of their life, such as HDPE [67], ABS [68], acrylonitrile styrene acrylate (ASA) [69], or PMMA [59], as mentioned, among others.

The amorphous nano-sized carbon allotrope of carbon black (CB) is characterized by high surface area thermal conductivity and electrical conductivity [70]. CB utilization can be effective for modifying the physical, mechanical, or electrical properties of the material selected for mixing [71]. This can enhance the conductivity, toughness, and weathering resistance of other matrix materials [72,73,74,75]. Applications such as servicing electronic devices, sensors, supercapacitor tires, and cable jackets are very common for CB [76], as are those of coloring pigments, printing ink ingredients, or adsorbent materials [77].

CB as a reinforcing filler [78] is a popular choice, and its market value has grown over the years, as indicated by existing related reports [79,80,81,82]. Grand View Research reported a potential 4.8% CAGR from 2024 to 2030, as USD 23,390 million (2024) could become USD 31,040 million (2030) [83]. A forecast analysis for the 2025–2033 period by IMARC projected a 3.57% CAGR from USD 17,900 million (2024) to USD 25,400 million (2033) [84]. There is also a report provided by Transparency Market Research indicating that the USD 18,600 million PMMA market volume in 2020 is expected to reach USD 29,900 million by 2031 (4.5% CAGR) [85].

Carbon black might not be the subject of a 3D printing investigation where PMMA is the matrix material, yet it has been combined with other polymers. For example, it has been part of a 3D printing-related investigation in which composites of polypropylene and CB were produced [86]. CB was also 3D printed along with PLA in separate investigations [87,88]. The ABS-CB composites were 3D printed and examined for their behavior [89,90]. In addition, efforts have been made to create polylactide/CB composites for fabricating 3D printed conductive products [91]. HDPE/CB [92] and polyamide/CB nanocomposites have been reported as well [93]. In all publications, virgin polymers were used (not recycled).

Based on the conducted bibliographic investigation, recycled PMMA is a viable material for MEX AM. Furthermore, CB has been reported to have reinforcing capabilities for other polymeric matrices. Yet, its efficacy in recycled PMMA thermoplastics for the MEX AM has not been reported, according to the research conducted in the bibliography. The impact of carbon black on polymer composites is not consistent; instead, it is heavily influenced by the characteristics of the host polymer [94]. The effects can vary between amorphous and semi-crystalline polymers [95]. Moreover, further examination is required to locate the optimal concentration of carbon black for specific polymers [96], while other parameters such as the polymer-filler interaction affect the behavior of the composites [97]. These aspects justify the need for such research.

In this research, nanocomposites of PMMA and CB were created with filler content of 0.0–12.0 wt.% (increased with a 2 wt.% step). The nanocomposites were first turned into the respective filaments for later utilization to produce the required coupons through a MEX 3D printing procedure. Subsequently, the microhardness (M-H), tensile and bending behavior, and impact strength (Charpy) of the coupons were investigated. The rheological, thermal, elemental, microstructural, and structural characteristics of the examined samples were also evaluated.

With regard to both tensile and bending behaviors, the strength, modulus of elasticity, and toughness were examined. Thermal evaluation was conducted by differential scanning calorimetry (DSC) and thermogravimetric analysis (TGA). The viscoelastic response was assessed with dynamic mechanical analysis (DMA). Viscosity and material flow rate (MFR) analyses were performed for rheological characterization, while the morphology of the 3D printed coupons from their lateral and fractured surfaces was revealed through scanning electron microscopy (SEM) images. The 3D printed (structural) quality was evaluated through micro-computed tomography (μ-CT), through the examination of the effect of CB on geometrical accuracy and the formation of the pores in the coupons.

The experiments provided significant information about the capabilities of recycled PMMA enhancement by the addition of the CB filler. PMMA/CB 4.0 wt.% was the nanocompound with the most enhanced result for tensile strength and tensile toughness, bending toughness, and filament tensile strength. Then PMMA/CB 6.0 wt.% presented the greatest results for tensile and bending modulus, while PMMA/CB 2.0 wt.% and PMMA/CB 12.0 wt.% were distinguished for their bending strength and M-H, respectively. The greatest dimensional deviation and porosity in relation to pure PMMA appeared to be in the case of 4.0 wt.% and 6.0 wt.% correspondingly. The utilization of recycled PMMA in nanocomposite formation in MEX AM further expands its sustainability and fields of application. Exploring the impact of the introduction of CB nanopowder in the recycled PMMA (thermoplastic) matrix led to the introduction of new PMMA/CB nanocomposites for the MEX AM method, with enhanced properties effective for the various types of applications PMMA is applied to.

## 2. Materials and Methods

**Figure 1:**
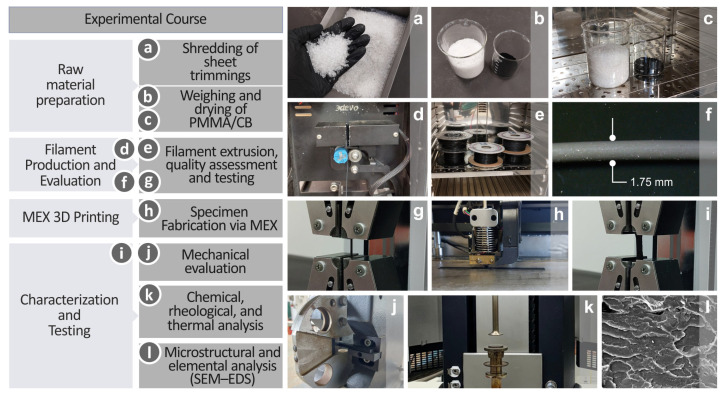
Experimental course presentation including, (**a**) PMMA sheet trimmings shredding, (**b**,**c**) PMMA and CB materials’ weighing and drying, (**d**–**g**) extrusion, quality assessment and testing of filaments, (**h**) coupon 3D printing via MEX, (**i**,**j**) mechanical testing, (**k**) chemical, rheological, thermal, and (**l**) microstructural and elemental examination.

The present research work followed an organized experimental course considering the materials in the original form (raw), filaments, and 3D printing, as well as experiments and evaluation processes, which are shown in Figure 1. With regard to the raw materials, shredding of sheet trimmings (Figure 1a), as well as PMMA/CB weighing and drying (Figure 1b,c), were conducted. Filament extrusion was accompanied by quality assessment and testing (Figure 1d–g). The filaments were then supplied for specimen fabrication via MEX (Figure 1h), and the produced coupons underwent mechanical evaluation (Figure 1i–j), chemical, rheological, and thermal testing (Figure 1k), as well as microstructural and elemental analyses (Figure 1l).

### 2.1. Materials

**Figure 2:**
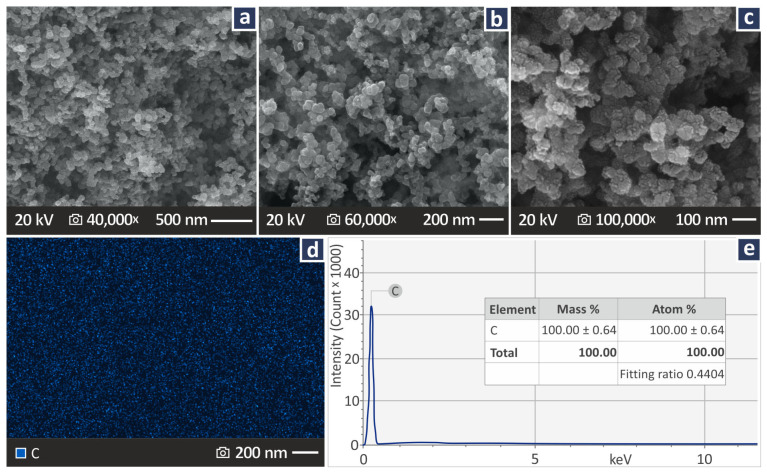
(**a**–**c**) CB SEM images at three different magnifications (40,000×, 60,000×, 100,000×), (**d**) C element dispersion, and (**e**) elemental chemical analysis of CB.

Plazcryl PMMA sheets purchased from Plazit Polygal Inc. (Charlotte, NC, USA) were used to create the final PMMA product suitable for filament extrusion. Sheets of 4 mm thickness were shredded to produce trimmings, which were then washed and dried properly. The CB filler material was supplied by Nanography, Ankara, Turkey, under the name Vulcan XC72 (surface area 254 m^2^/g, average particle size 30 nm, absorption 174 cc/100 g, Iodine number 253 mg/g, density 264 kg/m^3^, mesh residue < 25 ppm). Figure 2a–c presents the CB material SEM images at three different magnifications (40,000×, 60,000×, and 100,000×). Figure 2d shows the elemental C dispersion in the material, while Figure 2e shows the chemical analysis of the elements in the material. The images were taken within the context of the research with a field emission apparatus model named JSM-IT700HR by the company Jeol Ltd., established in Tokyo, Japan. The aim was to verify the size of the nanoparticles and evaluate their shape and elemental composition.

### 2.2. Mixtures, Filaments, and 3D Printed Coupon Preparation

Initially, the PMMA and CB materials were properly composed of mixtures of seven different filler percentages, namely, 0.0 wt.% to 12.0 wt.%, using a step of 2.0 wt.% (no other additives were used). The necessary amounts of materials were measured and prepared before being mixed by a blender operating at 4000 rpm for 20 min at high wattage, while their placement into a laboratory oven followed, with the aim of dehydration. The mixing took place in the blender at room temperature. This is the first step in contributing to the uniform dispersion of the filler in the matrix. Further mixing of the filler with the polymer in melting conditions was performed during the extrusion process to ensure the uniform dispersion of the filler in the matrix, which is crucial for the consistency and the performance of the prepared nanocomposites. This is presented further below.

The filler percentage range was decided based on the outcome of the preliminary experimental procedures. In particular, the performance of the composites was monitored, and the tests were terminated when there was no further improvement in their properties, indicating saturation of the nanoparticles [98,99], which was not desired in the current research, as it affects the mechanical performance in a negative way.

Melt extrusion of the filaments through 3D Evo Precision 450 (Utrecht, The Netherlands) was the next step, providing this research work with 1.75 mm filaments (±0.1 mm) of all the filler percentages created composites, which is a 3D printing accepted diameter. For the specimen fabrication, MEX AM Intamsys Funmat HT (Shanghai, China) was used, and coupons of five different types, namely tensile, bending, Charpy notched, DMA, and CT scan, were prepared (five per test and compound composition, following the respective test standards). In the supplementary material of this work, additional information related to filament diameter monitoring and inspection (Figure S1), as well as the specimen printing parameters and dimensions (Figure S2), is shown.

### 2.3. Raman Characteristics and Procedure

Raman spectra were acquired employing a LabRAM HR Raman Spectrometer by the HORIBA company, located in Kyoto, Japan.

- Excitation: solid-state laser module of 532 nm
- Maximum output power: 90 Mw
- Raman spectral resolution: ≈2 cm^−1^
- Grating with 600 grooves

Further information can be found in the supplementary file.

### 2.4. Rheological and Thermal Examination Procedure

For the thermal examination of the samples investigated herein, TGA and DSC analyses were conducted in the temperature range of 20–550 °C and 30–270 °C, respectively. TGA and DSC analyses were conducted under a nitrogen atmosphere, with gas flow rates of 200 mL/min and 50 mL/min and heating rates of 10 °C/min and 15 °C/min, respectively. The devices utilized to obtain the results were a Perkin Elmer Diamond TGA/DTGA (Waltham, MA, USA) and a model named DSC 25 by the company TA Instruments, with headquarters in New Castle, DE, USA.

For rheological characterization, a model named DHR-20 Discovery Hybrid Rotational Rheometer by the same company was employed, conducting viscosity tests. Attached to the apparatus, there was a parallel plate configuration characterized by a 25 mm diameter and an environmental test chamber with controllable temperature. The test was conducted in a controlled shear rate mode, with a gap of 1 mm between the plates. Each measurement point was recorded for 20 s, and accepted values were the ones within a 5% tolerance range. MFR tests were performed using a custom setup built according to the ASTM D1238-13 standard. A standard die was used (8 mm in height and 2.095 mm in diameter), and the applied load was 3.8 kg. The test was performed at the temperature instructed by the ASTM standard (230 °C). The setup allows the material to flow through the die so the melt flow rate can be measured in g/10 min.

### 2.5. Mechanical Testing Characteristics

Mechanical tests were performed to obtain data regarding the tensile, bending, Charpy notched impact, and microhardness. An Imada MX2 motorized test stand (Northbrook, IL, USA) was employed for the tensile and bending tests, whereas Charpy notched impact tests were performed employing a Terco MT220 (the Terco company headquarters are in Kungens, Sweden). For the tensile and bending investigations, proper grip equipment was used, while the following standards were used: ASTM D638-14, type V, 3.2 mm thickness, ASTM D790-10, and ASTM D6110 for tensile, bending, and Charpy tests, respectively. For Vickers M-H, the ASTM E384-17 was followed, and the employed machine was an Inova Test 300-Vickers apparatus from (Maastricht, The Netherlands).

Viscoelasticity was assessed on DMA on some of the 3D printed coupons based on the ASTM D4065-12 international standard. The data were obtained using a TA Instruments DHR20 device (New Castle, DE, USA), similar to the one used for the rheometric characteristics. The utilized temperature range was between 30 °C and 140 °C (5 °C/min temperature ramp, 0.1 N preload, and 30 μm constant oscillation amplitude at 1 Hz frequency).

### 2.6. Morphology and Structure Inspection

The morphological characterization of the coupons was performed using SEM analysis, which provided images of their lateral and fractured surfaces. The SEM field emission apparatus JSM-IT700HR, Jeol Ltd. (Tokyo, Japan), was used for this purpose, producing images of various magnifications. With regard to the structure of the coupons, the porosity and dimensional deviation were revealed with the assistance of micro-computed tomography (μ-CT), employing a Tomoscope HV Compact device, by the company Werth-Messtechnik GmbH, located in Gießen, Germany, to execute the analysis. Dimensional accuracy was evaluated using a 60 L resolution, whereas a 16 L resolution configuration was employed to examine the pores within the 3D printed structure. The μ-CT data were analyzed utilizing software from the company Volume Graphics GmbH, located in Heidelberg, Germany (VG Studio MAX, version 2.2). For these two-quality metrics (porosity and accuracy of the geometry), a sample was compiled by the authors, which is shown in the supplementary file. This featured different geometrical, prismatic, and curvature characteristics to include different aspects.

## 3. Results

### 3.1. Raman Evaluation

**Figure 3:**
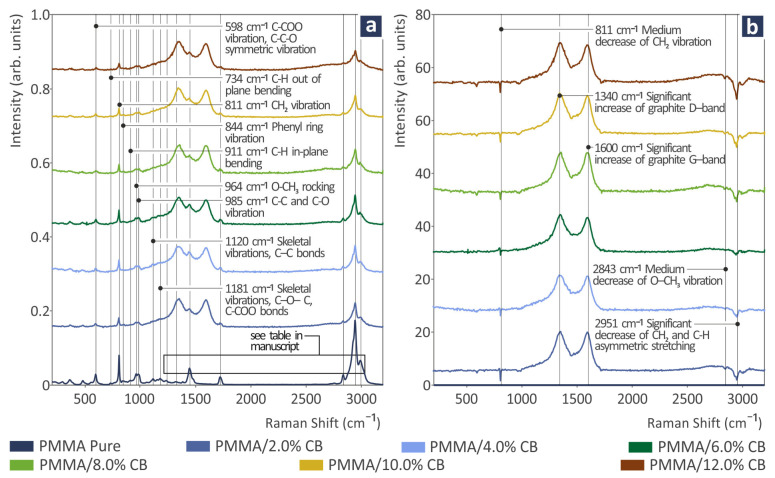
(**a**) Raman spectra from PMMA/CB 0.0–12.0 wt.%; (**b**) differences in the Raman spectra of PMMA/CB 2–12.0 wt.% from pure PMMA.

Figure 3a illustrates the Raman spectral profiles of the PMMA/CB mixtures. In the following Table 1, unfilled PMMA Raman peaks are depicted as they were reported in the literature together with their reference. As can be seen in Figure 3b, the addition of carbon black in PMMA presented a gradual intensity increase in the two graphite bands, the D-band at 1340 cm^−1^ and the G-band at 1590 cm^−1^ [100,101], which are commonly found in carbon, in all Raman spectra from PMMA/carbon black samples, as shown in Table 2, which proves the presence and gradual weight percentage increase. Moreover, a gradual decrease appeared linearly related to the weight percentage increase in carbon black within the samples at 811, 2843, and 2951 cm^−1^.

### 3.2. Rheology

**Figure 4:**
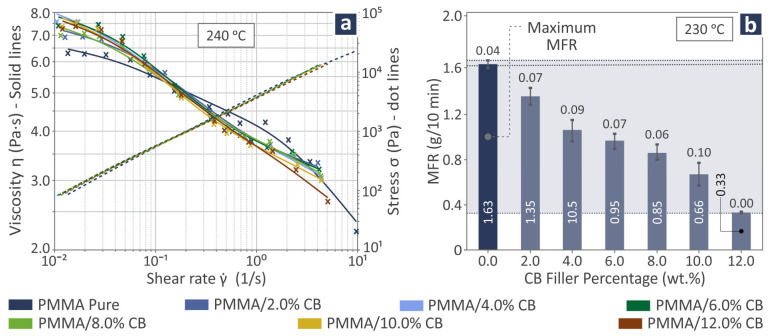
Rheological characteristics for pure PMMA and PMMA/CB (with filler percentage from 2.0 wt.% to 12.0 wt.%) in (**a**) profiles of stress vs. shear rate and viscosity vs. shear rate and (**b**) MFR versus CB filler percentage bars. The dark blue bar denotes the highest reported value.

The rheological characteristics of all PMMA/CB composite samples are shown in Figure 4 as stress vs. shear rate and viscosity vs. shear rate profiles at a temperature of 240 °C (Figure 4a), as well as the MFR levels in bars at 230 °C (Figure 4b). The graph indicates an increase in stress as the viscosity decreases, while the MFR bars show that as the filler percentage rises from 0.0 wt.% to 12.0 wt.%, the MFR levels lowered, with the pure PMMA having the greatest levels.

### 3.3. TGA/DSC/DMA Results

**Figure 5:**
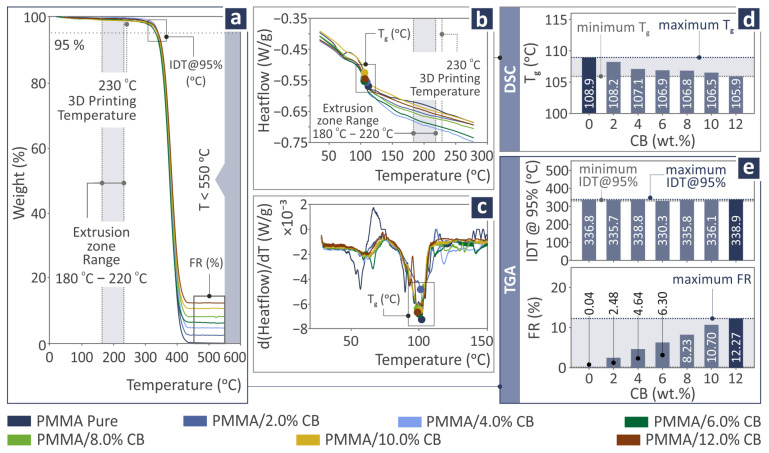
Thermal characteristics reveal for PMMA/CB (with filler percentage from 0.0 wt.% to 12.0 wt.%) in (**a**) TGA and (**b**) DSC curves, and possession of (**c**) first derivative of the DSC signal, (**d**) T_g_, and (**e**) IDT and FR recorded levels. The dark blue bar denotes the highest reported value.

The thermal characteristics of PMMA/CB (with filler percentage from 0.0 wt.% to 12.0 wt.%) are the subject of Figure 5. Figure 5a shows the TGA results for the weight percentage versus temperature curves, whereas Figure 5d presents the initial decomposition temperature (IDT), detecting a maximum in the case of 12.0 wt.% and final residue (FR), detecting a maximum again at 12.0 wt.%. Figure 5b depicts the first derivative of the DSC signal. As DSC curves are strongly overlapping around the temperature range including Tg, the first derivative of a DSC signal is provided, as it accentuates inflection points in the DSC curve, thereby enhancing the detection and resolution of closely spaced or overlapping thermal transitions, such as glass transitions, crystallizations, and melting events. Consequently, the first derivative can provide a more precise delineation of the onset, peak, and endset of thermal events. Therefore, it is presented to improve the comprehensiveness of the DSC findings. Figure 5c shows the DSC results for the heat flow as a function of temperature, whereas Figure 5d presents the glass transition temperature (T_g_) levels, with the maximum being at 0.0 wt.%.

**Figure 6:**
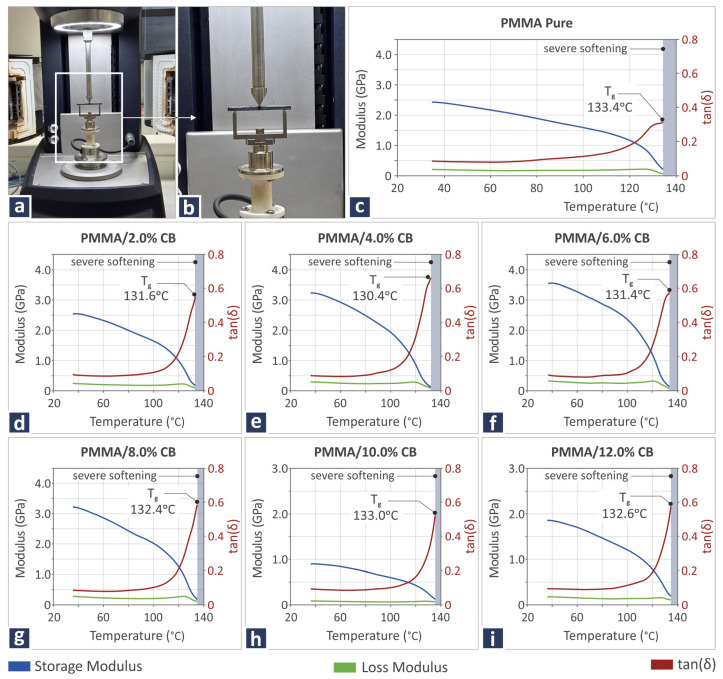
DMA-related information, including (**a**,**b**) DMA testing procedure, (**c**–**i**) storage modulus, loss modulus, and tan(δ) curves of PMMA/CB 0.0–12.0 wt.% samples.

The DMA results, revealing the viscoelastic behavior of the new nanocomposites prepared herein, are shown in Figure 6, including two images captured during the conduction of DMA (Figure 6a,b), as well as curves showing the storage and loss modulus, as well as tan(δ) for pure PMMA (Figure 6c), PMMA/CB 2.0 wt.% (Figure 6d), PMMA/CB 4.0 wt.% (Figure 6e), PMMA/CB 6.0 wt.% (Figure 6f), PMMA/CB 8.0 wt.% (Figure 6g), PMMA/CB 10.0 wt.% (Figure 6h), and PMMA/CB 12.0 wt.% (Figure 6i) samples. Notably, the CB introduction to PMMA led to a T_g_ temperature decrease in relation to pure PMMA, regardless of the CB filler percentage. Moreover, different storage modulus responses were observed for different filler quantities, particularly for the PMMA/CB 10.0 wt.%, which is also the composite sample presenting a different loss modulus behavior in relation to the rest of the composites.

### 3.4. Mechanical Performance at Room Temperature

**Figure 7:**
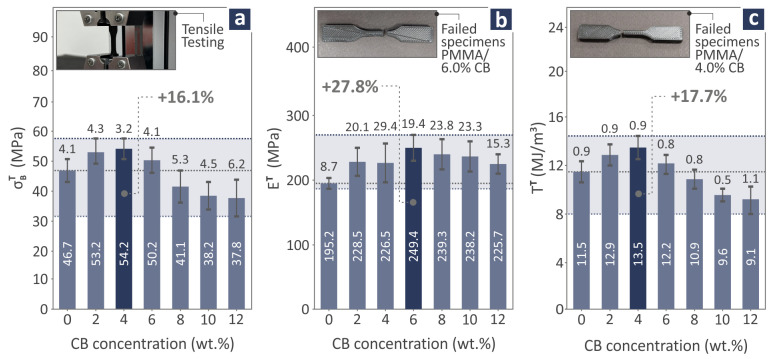
PMMA/CB 0.0–12.0 wt.% composite tensile coupon testing: (**a**) tensile strength levels and tensile testing image of a coupon, (**b**) tensile modulus of elasticity levels and a failed PMMA/CB 6.0 wt.% specimen image, and (**c**) tensile toughness levels and a PMMA/CB 4.0 wt.% specimen image. The dark blue bar denotes the highest reported value.

The mechanical behavior of the PMMA/CB samples was recorded through various mechanical tests related to tension, bending, Charpy impact, and M-H. The tension results of the PMMA/CB 0.0–12.0 wt.% coupon samples are depicted in Figure 7, showing the strength (Figure 7a), modulus of elasticity (Figure 7b), and toughness (Figure 7c) levels. Moreover, in Figure 7a–c there are three images depicting the tensile testing procedure: a failed PMMA/CB 6.0 wt.% specimen and a failed PMMA/CB 4.0 wt.% specimen, respectively. As can be observed, the PMMA/CB 4.0 wt.% levels were found to be 16.1% and 17.7% over pure PMMA, considering tensile strength and toughness correspondingly, while the tensile modulus of elasticity was measured to be 27.8% over pure PMMA, in the case of PMMA/CB 6.0 wt.% nanocompound.

**Figure 8:**
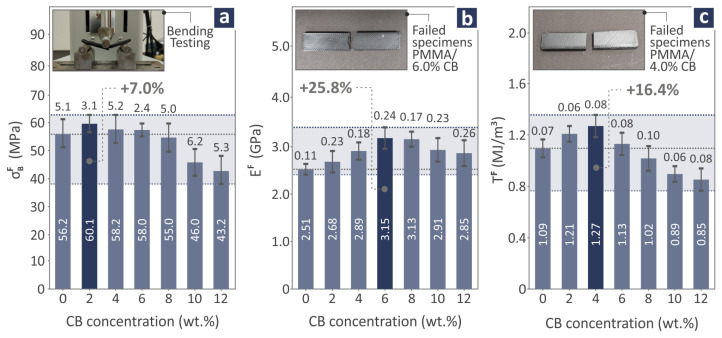
PMMA/CB 0.0–12.0 wt.% composite bending coupon testing: (**a**) bending strength levels and bending testing image of a coupon, (**b**) bending modulus of elasticity levels and a failed PMMA/CB 6.0 wt.% specimen image, and (**c**) bending toughness levels and a PMMA/CB 4.0 wt.% specimen image. The dark blue bar denotes the highest reported value.

Bending test results of PMMA/CB 0.0–12.0 wt.% coupon samples are depicted in Figure 8, showing the strength (Figure 8a), modulus of elasticity (Figure 8b), and toughness (Figure 8c) levels. In addition, Figure 8a–c exhibits three images of the tensile bending procedure, a failed PMMA/CB 6.0 wt.% specimen, and a failed PMMA/CB 4.0 wt.% specimen, respectively. PMMA/CB 2.0 wt.% levels were found to be 7.0% over pure PMMA at bending strength. PMMA/CB 6.0 wt.% was estimated to be 25.8% above pure PMMA for bending modulus of elasticity. The bending toughness of PMMA/CB 4.0 wt.% was 16.4% higher than pure PMMA.

**Figure 9:**
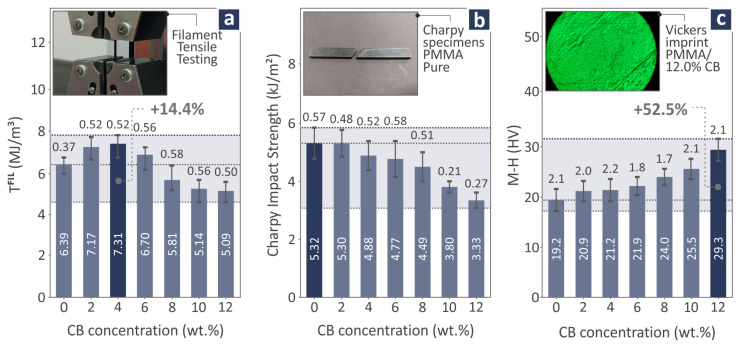
With regard to PMMA/CB 0.0–12.0 wt.% nanocomposites: (**a**) tensile toughness filament levels and tensile testing image of filament, (**b**) Charpy impact strength levels and a failed pure PMMA specimen image, and (**c**) M-H levels and a PMMA/CB 12.0 wt.% Vickers imprint image. The dark blue bar denotes the highest reported value.

In Figure 9a, the tensile toughness levels of PMMA/CB (0.0–12.0 wt.%) filaments are shown, out of which 4.0 wt.% is distinguished for its performance, exceeding pure PMMA by 14.4%. In the case of the Charpy impact strength levels (Figure 9b), pure PMMA seems to possess the highest levels, considering that as the filler concentration increases, the Charpy impact strength decreases. On the other hand, M-H increases as the filler quantity increases, resulting in a 12.0 wt.% increase in the PMMA/CB composite with the highest levels, exceeding pure PMMA by 52.5%. In Figure 9a–c there are three images showing the filament testing procedure, a failed Charpy pure PMMA specimen, and a PMMA/CB 12.0 wt.% Vickers imprint, respectively. Additional results regarding the mechanical characteristics of the PMMA/CB composite samples are presented in Figures S3 and S5 of the Supplementary Material.

### 3.5. Structural Performance

**Figure 10:**
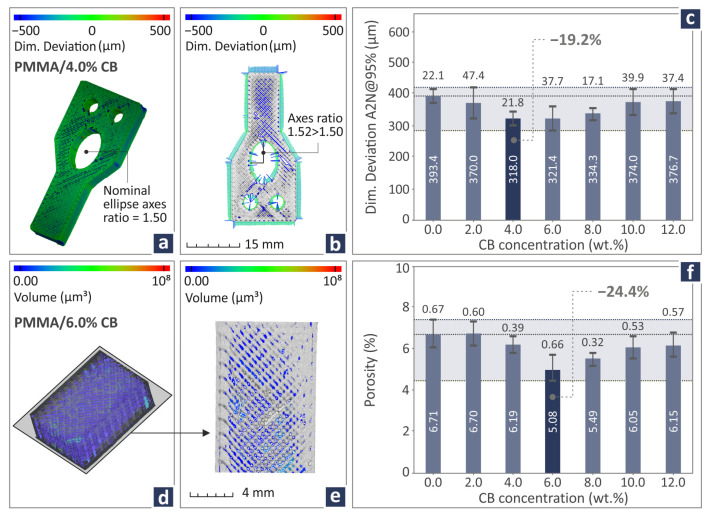
(**a**,**b**) PMMA/CB 4.0 wt.% dimensional deviation by color-coding mapping, (**c**) dimensional deviation levels of PMMA/CB (0.0–12.0 wt.%), (**d**,**e**) PMMA/CB 6.0 wt.% volume by color-coding mapping, (**f**) porosity levels of PMMA/CB composite samples. Dark blue bar indicates the best case reported.

Figure 10 shows the dimensional deviation and porosity-derived data from the examination of the PMMA/CB composites. In particular, Figure 10a,b show the dimensional accuracy of a PMMA/CB 4.0 wt.% CT-scan 3D printed sample by color-coding mapping. Figure 10c shows the dimensional deviation levels of all PMMA/CB (0.0–12.0 wt.%) composite samples and indicates that PMMA/CB 4.0 wt.% has the lowest dimensional deviation levels, 19.2% lower than pure PMMA. In Figure 10d,e, color-coding mapping is again applied for the projection of the PMMA/CB 6.0 wt.%. Figure 10f displays the porosity levels of all PMMA/CB (2.0–12.0 wt.%) nanocomposites and highlights the 6.0 wt.% as the one with the lowest porosity levels, being 24.4% lower than pure PMMA. Additional dimensional deviations and void graphs are presented in Figure S4 of the Supplementary Material.

### 3.6. SEM

**Figure 11:**
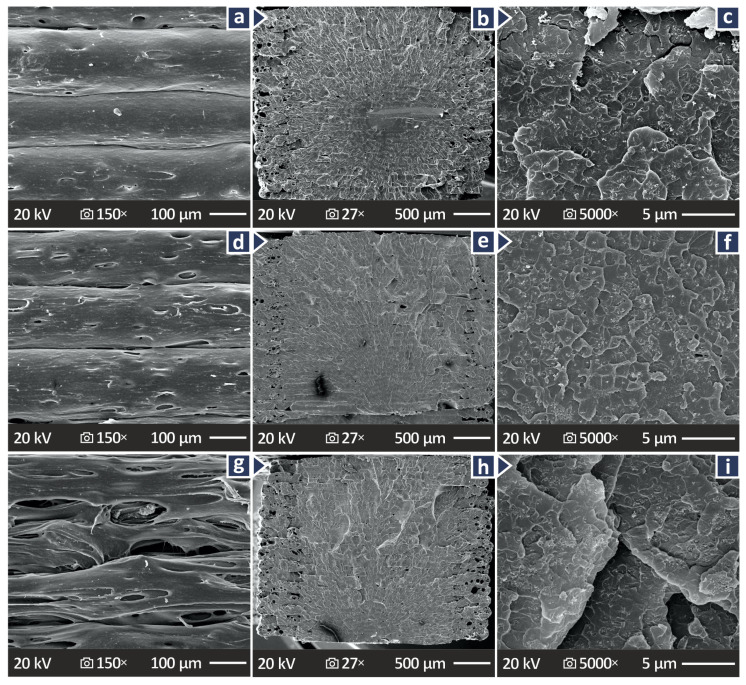
SEM images showing lateral surfaces in 150× magnification and fractured surfaces in 27× and 5000× magnification for (**a**–**c**) PMMA/CB 2.0 wt.%, (**d**–**f**) PMMA/CB 6.0 wt.%, and (**g**–**i**) PMMA/CB 10.0 wt.%, respectively.

**Figure 12:**
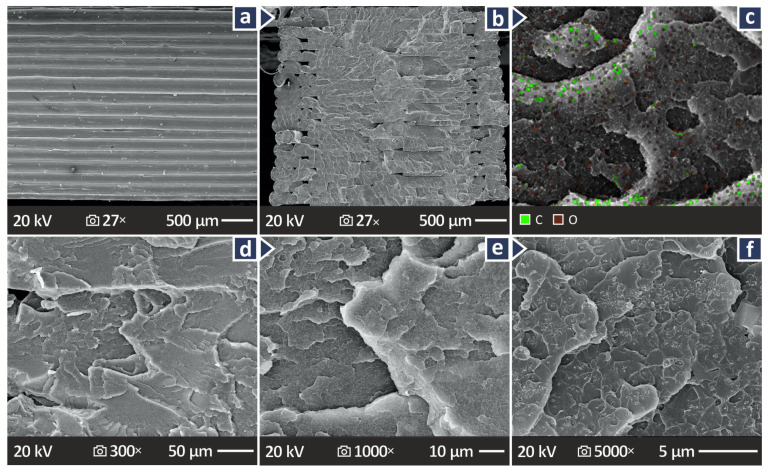
For PMMA/CB 12.0 wt.% (**a**) lateral surface in 27× magnification, (**b**) fractured surface in 27× magnification, and (**c**) EDS.

Figure 11 and Figure 12 present the SEM images captured showing the lateral and fractured surfaces of the coupons to study their microstructure and correlate this information with the data derived from the mechanical tests. With regard to the PMMA/CB 2.0 wt.%, PMMA/CB 6.0 wt.%, and PMMA/CB 10.0 wt.%,

Figure 11a,d,g show their lateral surface at 150× magnification.

Figure 11b,e,h show their fractured surfaces in 27× magnification, and

Figure 11c,f,i show their fractured surfaces at 5000× magnification. Some defects and voids can be detected when observing the lateral surfaces of the coupons, whereas layering in the case of 10.0 wt.% is uneven. As far as fractured surfaces are concerned, they appear to exhibit brittle behavior, with a few pores and voids.

Figure 12a shows a lateral surface SEM image of the PMMA/CB 12.0 wt.% composite sample (highest loaded sample), at 27× magnification, revealing a well-layered distribution, without defects. Figure 12b,d–f show the fracture surface images at 27×, 330×, 1000×, and 5000× magnification for the PMMA/CB 12.0 wt.% composite sample, revealing again a brittle behavior. Figure 12c shows an Energy-dispersive X-ray spectroscopy (EDS) image of the PMMA/CB 12.0 wt.% showing the dispersion of C and O elements in the material. The elements are distributed throughout the observation area, as expected.

## 4. Discussion

The objective of the present work is to examine the reinforcing abilities of CB on the behavior of recycled PMMA with regard to various aspects of its performance, including its mechanical properties and microstructure. The T_g_-derived values as part of the DSC thermal investigation showed maximum levels for pure PMMA (112.9 °C), which were reduced until PMMA/CB 4.0 wt.%, where the minimum was detected (105.5 °C), and then slightly began to increase again. The gathered T_g_ values from the DMA procedure show a similar pattern with DSC. However, T_g_ values derived from DMA were higher than those from DSC. The T_g_ derived through DMA should be considered more reliable. Furthermore, it should be noted that in the DMA examination, PMMA/CB 12.0 wt.% composites presented a different behavior than the rest of the composites considering storage and loss modulus. It should be noted that the maximum temperature used for the DMA tests was the temperature at which the samples were completely melted, but the usable measurements were until the temperature of “Severe softening”. This temperature is the temperature at which the sample becomes so soft that it deforms by its own weight and thus loses contact with the 3-point bending fixture, resulting in measurements that are unrepresentative of the materials’ behavior at those temperatures. The test did not stop at 140 °C; it continued normally. At that point, however, the specimen had already become very soft and was starting to lose contact with the fixtures, as shown by the near-zero values of the storage modulus. This is where the material can no longer sustain the measurement. Since the tan(δ) curves do not show clear peaks, T_g_ was assigned at the point where the storage modulus drops, and the sample loses mechanical stability.

Furthermore, in Figure 3 and Table 1 of Raman spectra of pure PMMA, a small peak at 844 cm^−1^ has been assigned to the phenyl ring, which is absent in the molecular structure of PMMA. The fact that it was measured indicates the presence of other materials such as crystalline polyethylene oxide (PEO), which is sometimes blended into PMMA materials [110,111], or styrene-methyl methacrylate (SMMA). This finding can be attributed to the fact that herein recycled PMMA from scrap was utilized as raw material. This scrap was taken from PMMA sheets. The PMMA matrix was not prepared by the authors from clear PMMA raw material. Since recycled PMMA sheets were used, the exact composition is not known. As mentioned, recycled PMMA from scrap as the matrix was used to prepare and evaluate fully sustainable composites in the current research.

The IDT values extracted after TGA were very close to each other for all the PMMA/CB composites, with the maximum located at 12.0 wt.% (338.9 °C) and the minimum at 6.0 wt.% (330.3 °C). Therefore, the introduction of CB in the recycled PMMA does not seem to affect the thermal stability of the thermoplastic. As far as final residue (FR) levels are concerned, they increased with the increase in CB, leaving the maximum at 12.0 wt.%. This is the expected outcome from these measures, as the CB increases in the nanocompounds.

Considering the mechanical behavior, the most interesting results were obtained for PMMA/CB 2.0, 4.0, 6.0, and 12.0 wt.% composite samples, which were distinguished for their great levels in one or more mechanical properties compared to pure PMMA. 2.0 wt.% presented a 7.0% improvement in bending strength, and 4.0 wt.% was the one affecting positively the most properties, including tensile strength (16.1%), tensile toughness (17.7%), bending toughness (16.4%), and filament tensile strength (14.4%). Then 6.0 wt.% revealed a considerable influence on tensile (27.8%) and bending (25.8%) modulus of elasticity. Finally, 12.0 wt.% projected a great improvement of 52.5% on M-H. It is worth mentioning that the Charpy impact strength decreased as a higher CB quantity was added to the composites.

From the above-reported findings, it can be noted that as the CB content increases in the nanocompounds, the toughness decreases. This means that the ability to absorb energy during the experimental process of the nanocompound is decreasing. This also is due to the increase in the brittleness of the nanocompounds with the increase of the CB content. Furthermore, the CB nanoparticles improved the stiffness of the nanocompounds up to a specific concentration (6 wt.%), which was the most prominent rise in their mechanical properties. On the other hand, the increase in stiffness did not converge with a strength increase. As stiffness (modulus) increases, ductility and ultimate strength can decrease due to crosslinking restricting polymer chain mobility, increasing stiffness [112] but making the material more brittle, reducing its elongation at break and strength in the tensile experiment [113]. This is the behavior found in these specific nanocompounds, which has been reported for other polymer-based materials before in the literature. In the bending test, the highest stiffness was found in the 6 wt.% nanocompound, while the highest flexural strength was reported in the 2 wt.% nanocompound and the toughness in the 4 wt.% nanocompound. This is a similar trend to the tensile experiment, showing again the increase in the brittleness of the samples. The impact strength was decreased by the addition of the CB nanoparticles. This can be attributed to the increase in the brittleness of the nanocompounds, which negatively affects the impact strength of polymeric materials [114]. On the other hand, the CB presence highly improved the M-H, with the improvement gradually increasing with the increase of the CB content in the nanocompounds. The 52.5% increase in the 12 wt.% nanocompound suggests that it is suitable for applications requiring high wear resistance from the materials.

Observing the dimensional deviation and porosity levels leads to the realization that again 4.0 wt.% and 6.0 wt.% were again those with the greatest results, 4.0 wt.% in the case of dimensional deviation (19.2% below pure PMMA) and 6.0 wt.% in the case of porosity (24.4% below pure PMMA). Consequently, it could be supposed that these filler concentrations were the most suitable for fabricating parts with enhanced properties and capabilities, even though other filler percentages could also be preferred and chosen depending on each application and its needs. Furthermore, the 6 wt.% nanocompound had the highest stiffness and good strength in both the tensile and bending experiments, showing a correlation between these metrics, with reduced porosity leading to higher mechanical performance. The 4.0 wt.% nanocompound was the one with the highest strength and the better dimensional accuracy, showing again that the print quality in the coupons is related and affects their mechanical response.

Microstructural investigation of the fabricated PMMA/CB 2.0, 6.0, 10.0, and 12.0 wt.% coupons revealed a considerably great amount of defects, voids, and pores in the case of 10.0 wt.%, which can be confirmed by correlating the SEM images with the respective dimensional deviation and porosity levels. The rheological measurements revealed an MFR decrease due to the addition of the CB filler and further decreased as the CB content was raised in the nanocompounds. This means that the melted resin flows less easily, and processing becomes more difficult. Therefore, the reduced 3D printing quality observed in SEM can be attributed to this influence of the CB nanopowder on the rheology of the recycled PMMA. Adjustments to the 3D printing settings are required to overcome this issue. However, this was not completed in the research to ensure the reliability of the experimental findings’ comparison. Overall, all the samples undergoing SEM indicated brittle behavior and good material dispersion during the 3D printing process, except for 10.0 wt.%, which was slightly uneven.

It should be noted that the MFR temperature in the measurements was the temperature required to perform the test in accordance with the respective standard. Regarding the viscosity test, the maximum temperature is selected according to the applied 3D printing temperature. Still, a temperature of 240 °C was chosen, 10 °C higher than the applied 3D printing temperature. Although 230 °C is the 3D printing temperature, 240 °C is still within the material’s processing range, and this would help to better understand how the material behaves under slightly higher thermal and shear conditions during the extrusion process.

Furthermore, in the rheology measurements, a slight increase in shear viscosity for the filled system compared to the unfilled one was observed. At such a high filler content, particle-particle interactions start to develop, which explains the increase in viscosity. However, this effect remains relatively moderate, which is typical in systems with this kind of filler and dispersion. This small increase is expected and does not necessarily indicate slippage or deviation from the flow regime.

As the bibliographic research showed, no similar nanocompounds have been introduced yet to directly correlate the reported outcome. Still, the results can be partially compared with correlated publications. Research with PMMA polymer and respective nanocompounds prepared with a similar thermomechanical method for the MEX AM technique is considered. The materials preparation method and the parts fabrication method highly affect their performance. Therefore, comparisons with materials prepared with different techniques or for use in different manufacturing processes could be misleading and unreliable. The strength of the unfilled recycled PMMA reported herein is in very good agreement with the strength of virgin PMMA for parts made with the MEX AM method [53]. This shows that PMMA can be used in recycled form in MEX AM without deuteration of its mechanical properties. Furthermore, research on the reprocessing of PMMA in MEX AM has shown that the remaining properties of the thermoplastic are also not compromised by the reprocessing method, at least for six successive thermal courses [59]. Regarding the CB filler, its reinforcement capabilities regarding the tensile strength were similar to the current research on the PLA [88] and polyamide [93] nanocomposites. Still, the improvement in stiffness was much higher in the recycled PMMA compared to the other two polymers. It should be noted that in the other two polymers, the highest mechanical performance was achieved with lower CB content in the nanocompounds than in the current research. On the other hand, the reinforcement capabilities of CB were much better on the HDPE polymer [92]. In this case, the highest mechanical performance was achieved with much higher CB content in the nanocompounds than in the current research. These differences are expected due to the different interactions between the matrices and the filler, the varied experimental conditions, and the different CB nanoparticle grades. Such differences justify the need for individual research for each matrix/filler combination, while the recycled matrix employed herein is an additional challenge.

## 5. Conclusions

Herein, the possibility of using recycled PMMA as a matrix material for nanocomposites in MEX AM was explored. The aim was to enhance the sustainability of the material by enabling new usage of it after its end of life. Furthermore, the development of nanocomposites with it as a matrix aimed at proposing new materials for MEX AM with improved characteristics and performance. CB was evaluated as a reinforcement additive for this purpose. The feasibility of creating PMMA/CB nanocomposites with a thermomechanical method compatible with MEX AM was explored, evaluating parameters such as the processability and the CB filler percentage, achieving better results in the thorough characterization process conducted, including different types of properties and parameters. Following this approach, it was achieved to provide the research community with valuable information based on the results derived. Raw materials were extruded into filaments, which underwent inspection and testing before moving on to supplying the 3D printing procedure of a specimen series. The fabricated coupons were examined and evaluated for their rheology, thermal properties, mechanical characteristics, microstructure, and elemental composition. PMMA/CB 4.0 wt.% composite samples revealed improvement in many of the properties considered (tensile strength and toughness, bending toughness, filament tensile strength, and dimensional deviation) in relation to composite samples of different filler percentages. Thus, this should be considered the optimum loading for the introduced recycled PMMA/CB nanocomposites. In particular, the best mechanical properties were those of tensile toughness, projecting a 17.7% increase in relation to pure PMMA. On the other hand, the 19.2% reduction in the dimensional deviation for 4.0 wt.%, in comparison with pure PMMA was also remarkable. The most notable improvement was the increase in the stiffness of the nanocompounds, while this came with an increase in their brittleness as well. PMMA/CB 6.0 wt.% was a composite that indicated greater improvement of these properties. Tensile and bending moduli of elasticity of 6.0 wt.% were found to be 27.8% and 25.8% above pure PMMA. Unrivaled were the M-H results of PMMA/CB 12.0 wt.%, which projected a 52.5% increase over pure PMMA, showing potential for use in high wear environments. When strength and toughness are the priorities, the 4 wt.% nanocompound is better; when stiffness is the priority, the 6 wt.% responds in a better way; and when wear resistance is the main specification, the 12 wt.% is more suitable.

Overall, CB enhanced recycled PMMA’s performance and improved the quality metrics of the 3D printed parts, leading this study to fill a research gap and create new opportunities for various potential applications in different fields. The results strengthen the sustainability of the PMMA material. This finding leads to future research toward the evaluation of alternative fillers that will make the prepared nanocompound suitable and more focused on other types of applications. PMMA is used and can be exploited after the end of its life. Furthermore, future work could include the optimization of the 3D printing parameters, considering the noteworthy impact of the CB nanoparticles on the rheology of the recycled PMMA thermoplastic.

## Figures and Tables

**Table 1:** Raman peaks (significant) and their respective assignments from pure PMMA.

Wavenumber (cm^−1^)	Intensity	Raman Peak Assignment
598	Strong	C-COO vibration, C-C-O symmetric vibration [102]
734	Small	C-H out-of-plane bending [103,104]
811	Strong	CH_2_ vibration [102]
844	Small	Phenyl ring vibration [105]
911	Small	C-H in-plane bending [104]
964	Strong	O-CH_3_ rocking [102]
985	Strong	C-C and C-O vibration [106]
1120	Medium	Skeletal vibrations, C–C bonds [102,106]
1181	Small	Skeletal vibrations, C–O– C, C-COO bonds [102,103,106]
1240	Small	C-O-C stretching [104]
1326	Small	C-O-C stretching [104]
1449	Strong	C-H_3_ deformation [102,103,104]; C-H_2_ deformation [103,104]; C-H_3_ symmetric bending [104,105,107];
1727	Strong	C = O bond [102,108] C-O-C symmetric stretching [109]
2843	Medium	O-CH_3_ vibration [102]
2951	Strong	CH_2_ and C-H asymmetric stretching [102,106]
3000	Medium	C-H stretching [104]

**Table 2:** Raman peak (significant) variations of PMMA/CB samples from pure PMMA.

811 cm^−1^	Gradual decrease	Medium decrease of CH_2_ vibration
1340 cm^−1^	Gradual increase	Significant increase of graphite D-band
1600 cm^−1^	Gradual increase	Significant increase of graphite G-band
2843 cm^−1^	Gradual decrease	Medium decrease of O-CH_3_ vibration
2951 cm^−1^	Gradual decrease	Significant decrease of CH_2_ and C-H asymmetric stretching

## Data Availability

The raw/processed data required to reproduce these findings cannot be shared because of technical or time limitations.

## Supplementary Materials

The following supporting information can be downloaded at: https://www.mdpi.com/article/10.3390/polym17101383/s1, Figure S1. (a) Pure PMMA filament diameter monitoring and inspection, (b) PMMA/CB (0.0–12.0 wt.%) filaments tensile strength levels, (c) PMMA/CB 6.0 wt.% filament diameter monitoring and inspection, (d) PMMA/CB (0.0–12.0 wt.%) filaments tensile modulus of elasticity levels; Figure S2. Specimen 3D printing parameters and possession of the models and dimensions of all the specimen types, taken into consideration; Figure S3. With regard to PMMA/CB (0.0–12.0 wt.%) samples, (a) filament tensile stress to strain curves, (b) specimen tensile stress to strain curves, (c) specimen flexural stress to strain curves, and the respective image captured during each testing; Figure S4. Graphs presenting (a) the relative surface and deviating point versus the dimensional deviation, (b) the void volume versus void diameter, and (c) void compactness and void sphericity versus void diameter of all the PMMA/CB composite samples and pure PMMA; Figure S5. Concentric spider graphs showing (a) the tensile strength, (b) flexural strength, (c) dimensional deviation, and (d) porosity of all the PMMA/CB composite tested specimen samples.
