# Supplementary material for: Valorization of Polymethylmethacrylate Scrap Reinforced with Nano Carbon Black with Optimized Ratio in Extrusion-Based Additive Manufacturing
Source: Polymers (Basel). 2025 May 17;17(10):1383. doi: 10.3390/polym17101383 (PMC12114872; doi:10.3390/polym17101383)
Supplement: Supplementary file 1 [file polymers-17-01383-s001.zip › polymers-3618053-supplementary.pdf]

polymers

MDPI

Article

Valorization of Polymethylmethacrylate Scrap Reinforced with Nano Carbon Black with Optimized Ratio in Extrusion-Based Additive Manufacturing

Nikolaos Michailidis [1,2], Nectarios Vidakis [3], Constantine David [4], Dimitrios Sagris [4], Vassilis M. Papadakis [5,6], Apostolos Argyros [1,2], Nikolaos Mountakis [3], Maria Spyridaki [3] and Markos Petousis [3,*]

[1] Physical Metallurgy Laboratory, Mechanical Engineering Department, School of Engineering, Aristotle University of Thessaloniki, 54124 Thessaloniki, Greece; nmichail@auth.gr (N.M.); aargyros@auth.gr (A.A.)
[2] Centre for Research & Development of Advanced Materials (CERDAM), Centre for Interdisciplinary Research and Innovation, Balkan Centre, Building B', 10th km Thessaloniki-Thermi road, 57001 Thessaloniki, Greece
[3] Department of Mechanical Engineering, Hellenic Mediterranean University, 71410 Heraklion, Greece; vidakis@hmu.gr (N.V.); mountakis@hmu.gr (N.M.); mspyridaki@hmu.gr (M.S.)
[4] Department of Mechanical Engineering, International Hellenic University, Serres Campus, 62124 Serres, Greece; david@ihu.gr (C.D.); dsagris@ihu.gr (D.S.)
[5] Department of Industrial Design and Production Engineering, University of West Attica, 12243 Athens, Greece; v.papadakis@uniwa.gr
[6] Institute of Electronic Structure and Laser of the Foundation for Research and Technology-Hellas (IESL-FORTH)–Hellas, N. Plastira 100m, 70013 Heraklion, Greece
* Correspondence: markospetousis@hmu.gr; Tel.: +30-2810379227

Academic Editor(s): Name

Received: 15 April 2025
Revised: 14 May 2025
Accepted: 15 May 2025
Published: date

Citation: Michailidis, N.; Vidakis, N.; David, C.; Sagris, D.; Papadakis, V.M.; Argyros, A.; Mountakis, N.; Spyridaki, M.; Petousis, M. Valorization of Polymethylmethacrylate Scrap Reinforced with Nano Carbon Black with Optimized Ratio in Extrusion-Based Additive Manufacturing. *Polymers* **2025**, *17*, x. https://doi.org/10.3390/xxxxx

Copyright: © 2025 by the authors. Submitted for possible open access publication under the terms and conditions of the Creative Commons Attribution (CC BY) license (https://creativecommons.org/licenses/by/4.0/).

Abstract: To promote environmental sustainability, this research investigated the potential of utilizing recycled polymethylmethacrylate (PMMA) as raw material in material extrusion (MEX) additive manufacturing (AM). To enhance its mechanical response, carbon black (CB) was employed as the filler in nanocomposite formation. Filament extrusion of the mixture at different concentrations produced printable feedstocks for MEX AM. Rheological analysis (viscosity and material flow rate) showed that the CB introduction to the matrix was beneficial for consistent layer deposition, while differential scanning calorimetry and thermogravimetric analyses verified the thermal stability of the nanocomposites during processing. Mechanical properties were optimized, with increases in modulus (27.8% and 25.8% respectively in tensile and bending loadings) and tensile strength at optimal CB loadings. Dynamic mechanical analysis revealed the viscoelastic response of the nanocomposites. Raman and energy dispersive spectroscopy provided element-related insights. Surface morphology and parts structure were observed employing scanning electron microscopy and micro-computed tomography respectively, revealing a positive impact on the AM parts due to the CB presence in the nanocomposites. The 4 wt.% in CB content nanocomposite was the optimum one. This research pioneers the development of new sustainable nanocomposite filaments and highlights the potential of next-generation MEX-based AM.

Keywords: Polymethylmethacrylate (PMMA); Carbon Black (CB); Three-Dimensional (3D) Printing; Environmental sustainability; material extrusion; recycling; mechanical characterization; additive manufacturing

S.1. Filament evaluation

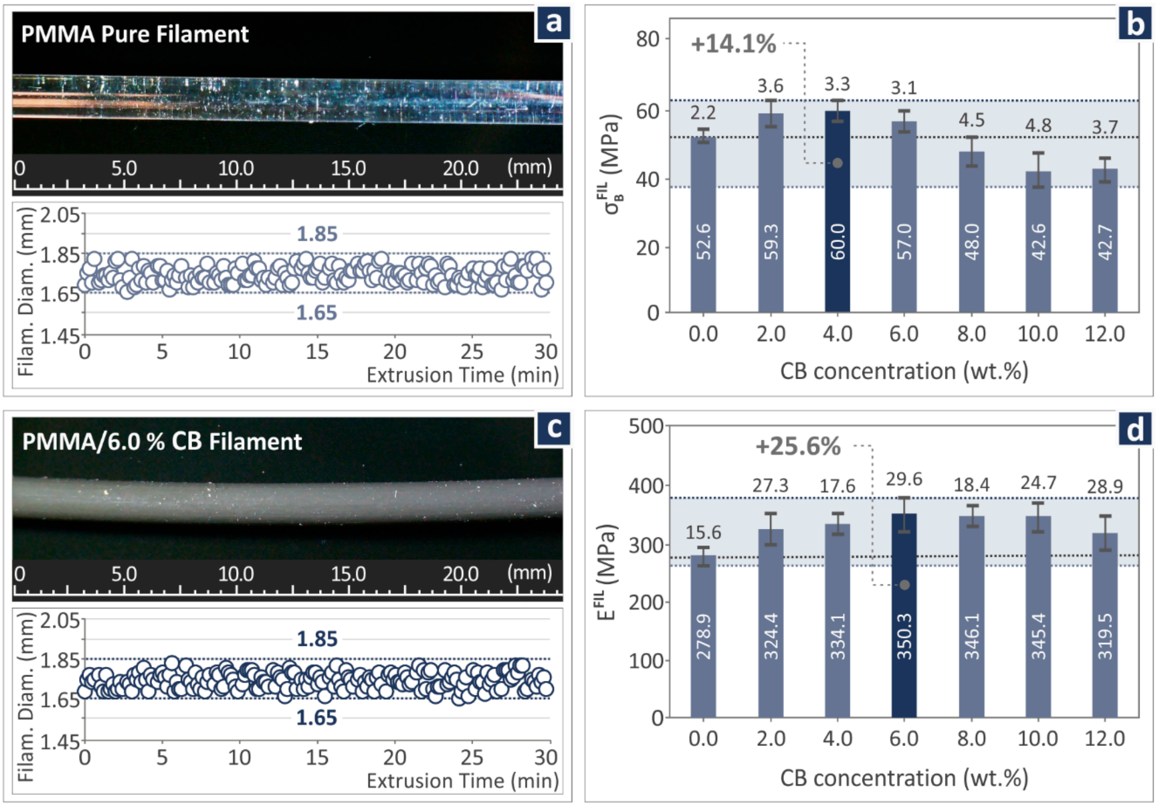

Figure S1. (a) PMMA pure filament diameter monitoring and inspection, (b) PMMA/CB (0.0-12.0 wt.%) filaments tensile strength levels, (c) PMMA/CB 6.0 wt. % filament diameter monitoring and inspection, (d) PMMA/CB (0.0-12.0 wt.%) filaments tensile modulus of elasticity levels.

S.2. Raman

Raman spectra were acquired using a HORIBA LabRAM HR Raman Spectrometer (Kyoto, Japan).

- Excitation: 532 nm solid-state laser module
- Maximum output power: 90 Mw
- Raman spectral resolution: ≈2 cm-1
- Grating with 600 grooves

Light was projected onto the samples using an Olympus objective lens (LMPlanFL N) (numerical aperture: 0.5) and Raman signals were collected. The working distance of the objective lens with 50× magnification was 10.6 mm. A Neutral Density filter with 3.2% transmittance was responsible for the laser power limitation (recorded to be 1.2 mW on the sample). The lateral and axial measurement volume were 1.7 μm and axial was 2 μm, respectively. The three optical windows contributed to the collection of the Raman spectra (between 40 and 3900 cm-1). There was 30 s of exposure for each point and five accumulations. Discoloration and degradation due to laser irradiation were prevented by visual inspection of the irradiated areas.

The HORIBA LabSpec software (Kyoto, Japan) was used for raw Raman data processing. With regard to the acquired spectrum processing, the following methodology was used: a) Cosmic ray removal; b) signal denoising (5 points kernel); c) data cropping between the range of 200 and 3200 cm^{-1}; d) background removal (8th grade polynomial utilization); e) Spectra recalibration by the 810 cm-1 peak; f) Spectral normalization by the unit vector function.

S.3. 3D printing parameters and coupons dimensions

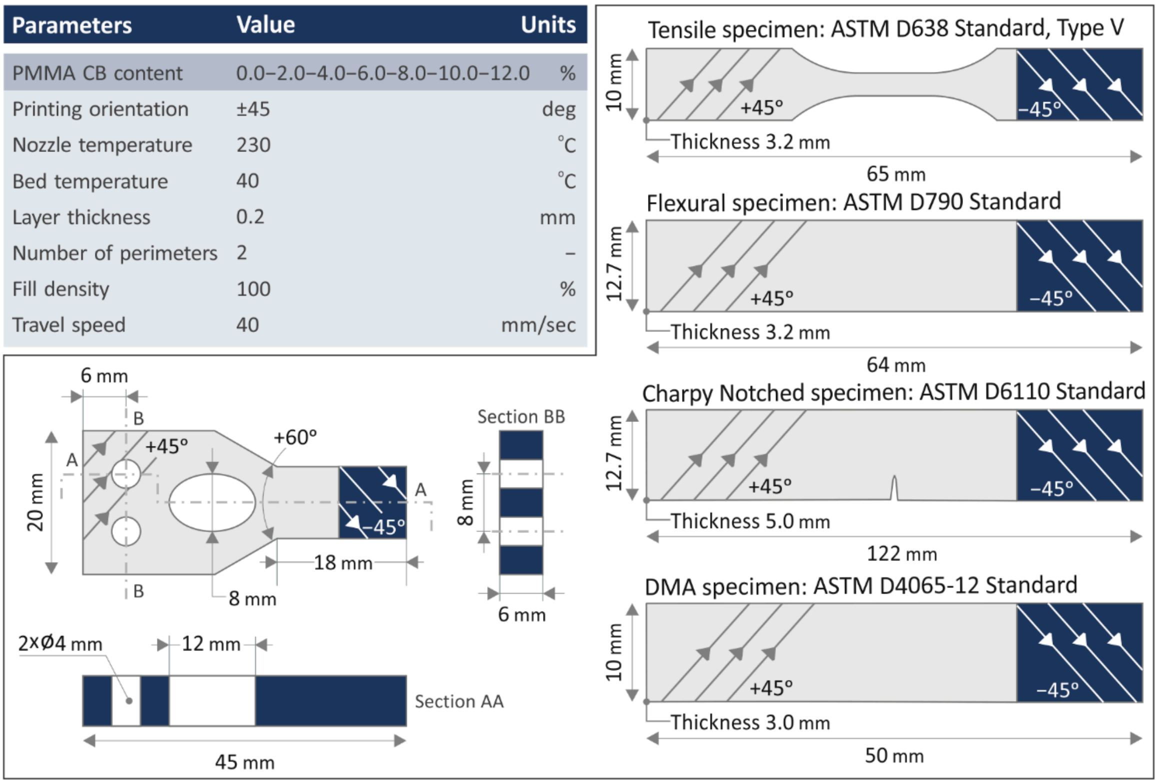

Parameters	Value	Units
PMMA CB content	0.0–2.0–4.0–6.0–8.0–10.0–12.0	%
Printing orientation	±45	deg
Nozzle temperature	230	°C
Bed temperature	40	°C
Layer thickness	0.2	mm
Number of perimeters	2	–
Fill density	100	%
Travel speed	40	mm/sec

Figure S2. Specimen 3D printing parameters and possession of the models and dimensions of all the specimen types, taken into consideration.

S.4. Stress vs. strain graphs

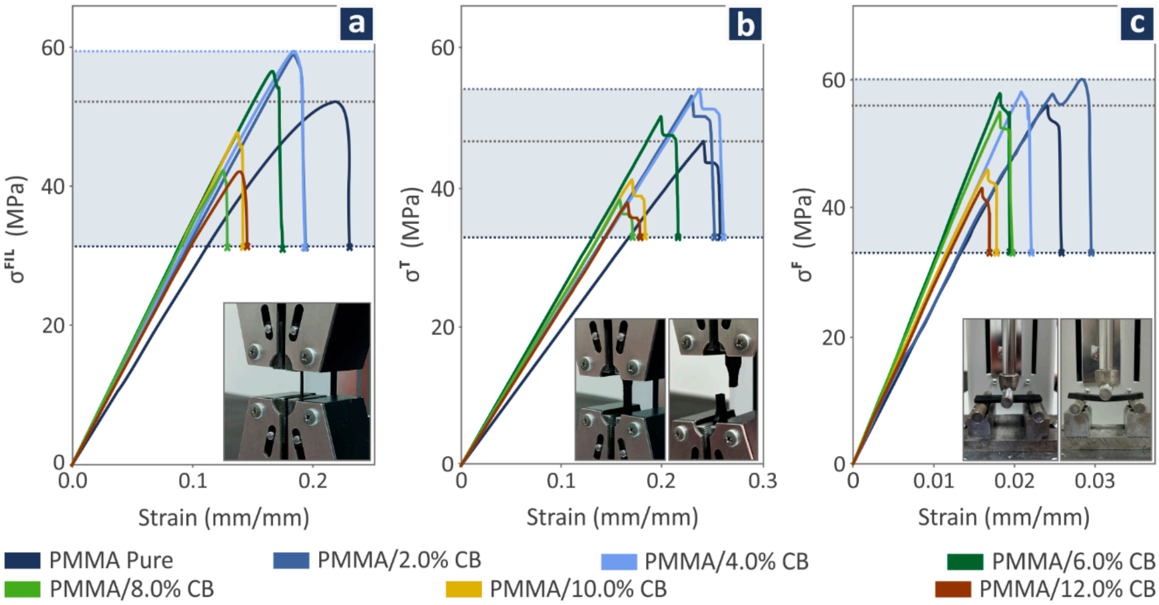

Figure S3. With regard to PMMA/CB (0.0-12.0 wt. %) samples, (a) filament tensile stress to strain curves, (b) specimen tensile stress to strain curves, (c) specimen flexural stress to strain curves and the respective image captured during each testing.

S.5. μ-CT results

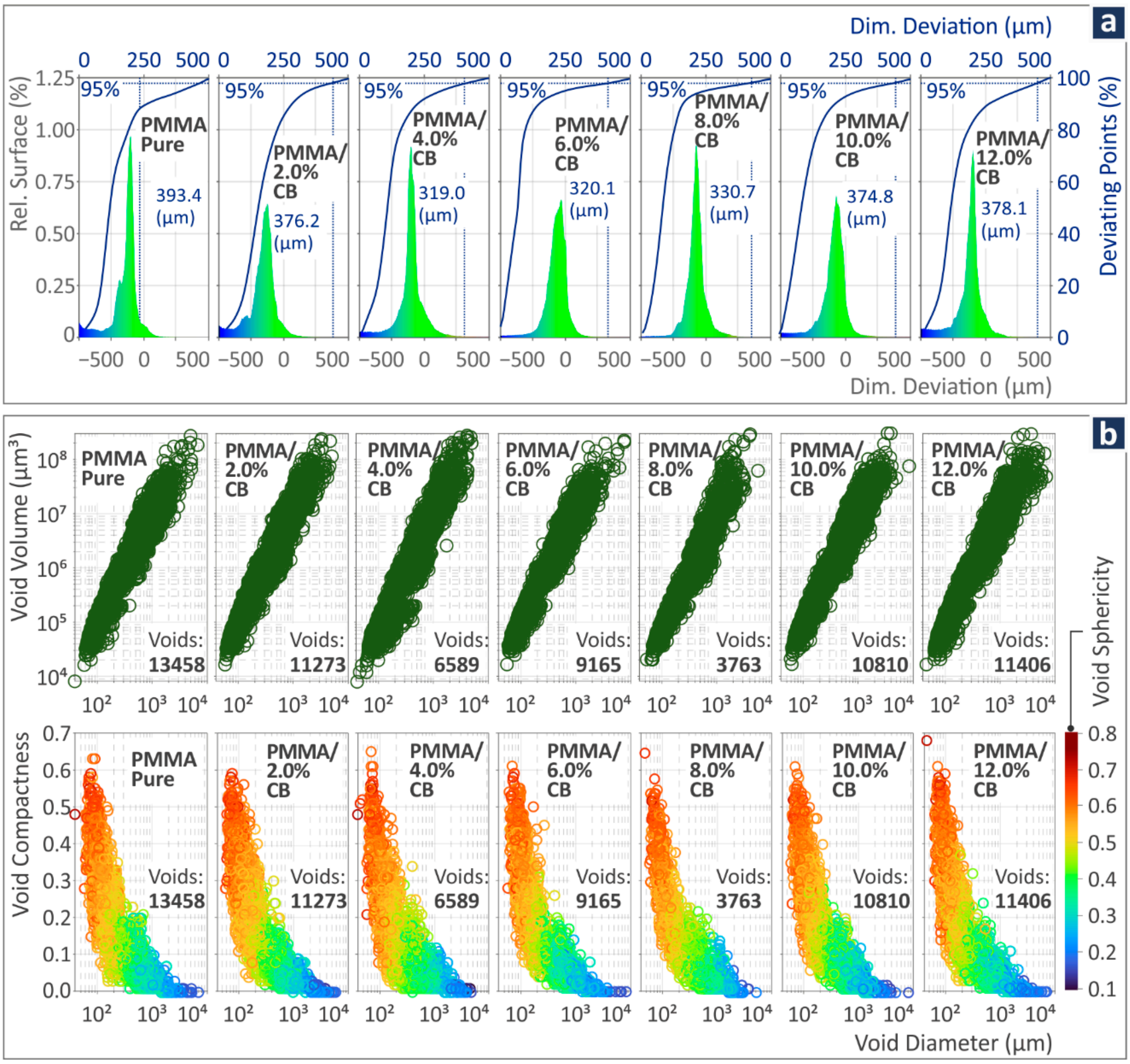

Figure S4. Graphs presenting (a) the relative surface and deviating point versus the dimensional deviation, (b) the void volume versus void diameter, (c) void compactness and void sphericity versus void diameter of all the PMMA/CB composite samples and PMMA pure.

S.6. Experimental results summary

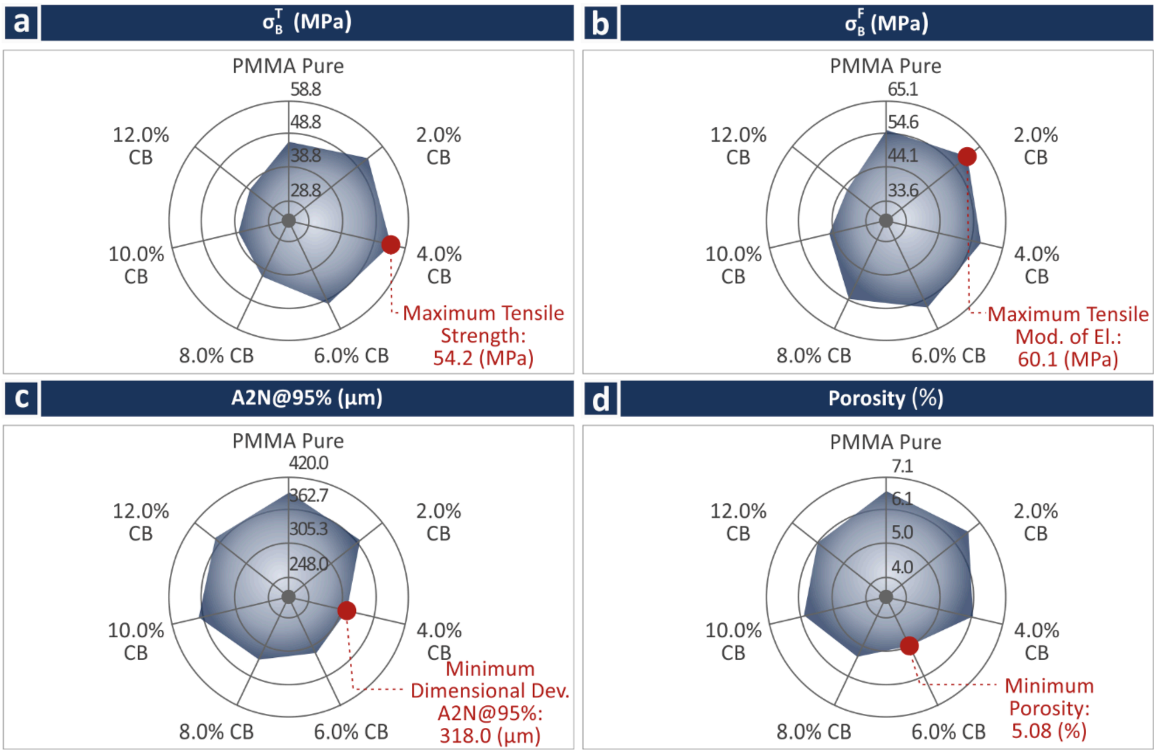

Figure S5. Concentric spider graphs showing (a) the tensile strength, (b) flexural strength, (c) dimensional deviation and (d) porosity of all the PMMA/CB composite tested specimen samples.

Disclaimer/Publisher's Note: The statements, opinions and data contained in all publications are solely those of the individual author(s) and contributor(s) and not of MDPI and/or the editor(s). MDPI and/or the editor(s) disclaim responsibility for any injury to people or property resulting from any ideas, methods, instructions or products referred to in the content.
